# Efficient Noninvasive FHB Estimation using RGB Images from a Novel Multiyear, Multirater Dataset

**DOI:** 10.34133/plantphenomics.0068

**Published:** 2023-07-14

**Authors:** Dominik Rößle, Lukas Prey, Ludwig Ramgraber, Anja Hanemann, Daniel Cremers, Patrick Ole Noack, Torsten Schön

**Affiliations:** ^1^ AImotion Bavaria, Technische Hochschule Ingolstadt, Ingolstadt, Germany.; ^2^ Hochschule Weihenstephan-Triesdorf, Weidenbach, Germany.; ^3^ Saatzucht Josef Breun GmbH and Co. KG, Herzogenaurach, Germany.; ^4^ Technical University of Munich, Munich, Germany.

## Abstract

*Fusarium* head blight (FHB) is one of the most prevalent wheat diseases, causing substantial yield losses and health risks. Efficient phenotyping of FHB is crucial for accelerating resistance breeding, but currently used methods are time-consuming and expensive. The present article suggests a noninvasive classification model for FHB severity estimation using red–green–blue (RGB) images, without requiring extensive preprocessing. The model accepts images taken from consumer-grade, low-cost RGB cameras and classifies the FHB severity into 6 ordinal levels. In addition, we introduce a novel dataset consisting of around 3,000 images from 3 different years (2020, 2021, and 2022) and 2 FHB severity assessments per image from independent raters. We used a pretrained EfficientNet (size b0), redesigned as a regression model. The results demonstrate that the interrater reliability (Cohen’s kappa, *κ*) is substantially lower than the achieved individual network-to-rater results, e.g., 0.68 and 0.76 for the data captured in 2020, respectively. The model shows a generalization effect when trained with data from multiple years and tested on data from an independent year. Thus, using the images from 2020 and 2021 for training and 2022 for testing, we improved the $F_{1}^{w}$ score by 0.14, the accuracy by 0.11, *κ* by 0.12, and reduced the root mean squared error by 0.5 compared to the best network trained only on a single year’s data. The proposed lightweight model and methods could be deployed on mobile devices to automatically and objectively assess FHB severity with images from low-cost RGB cameras. The source code and the dataset are available at https://github.com/cvims/FHB_classification.

## Introduction

Worldwide, *Fusarium* head blight (FHB), also called wheat scab or ear blight, is the most prevalent floral disease in wheat (*Triticum aestivum*) [1–3]. Besides causing substantial yield losses and decreasing baking quality, FHB is a major source of mycotoxins in grains. In particular, the dominant mycotoxin deoxynivalenol constitutes important health risks [1,2]. FHB is caused by various Ascomycete *Fusarium* species such as *Fusarium culmorum* and *Fusarium graminearum*. The main FHB symptom is the whitening of spikelets up to the entire ear. Depending on the weather conditions, the complete colonization of the ear can take approximately 10 to 14 days [1].

FHB was predicted to profit from ongoing climatic change due to shifted wheat flowering, drought stress of the host plant, and temperature increase [4]. Therefore, resistance breeding needs to be further intensified. Commonly, this process involves testing of thousands of breeding lines under field conditions and often includes artificial inoculation [1,4]. Therefore, there is an urgent need for efficient phenotyping of FHB under field conditions.

Biochemical methods are available for detecting mycotoxins produced from FHB but remain time-consuming and expensive [2]. Hitherto, field-based scoring of FHB severity relies on visual assessment of the disease incidence and severity on the wheat’s ear [5], being time-consuming, expensive, and subjective, depending on the breeders‘ perception and experience [6]. Consequently, FHB scoring is limited to a few points in time, thus limiting the monitoring of the infestation over time and the comparison of phenologically shifted genotypes. Therefore, sensor-based high-throughput phenotyping of FHB could facilitate the scoring process.

Previous approaches estimated FHB severity ex situ using hyperspectral data [7–10]. While hyperspectral imaging achieved good detection of FHB also in the field [11–13], this approach cannot be readily utilized for high-throughput field-based phenotyping due to the long measurement time and high sensor costs [14]. Unlike hyperspectral cameras, red–green–blue (RGB) imaging is characterized by low sensor costs, simple sensor handling, and fast scanning but is limited to the visible spectrum.

In recent years, deep learning methods have brought substantial progress to image-based plant disease detection [15]. Using images of dissected ears, Gao et al. [16] applied transfer learning based on networks pretrained on the ImageNet dataset [17] for the prediction of visual FHB scores, recommending the ResNet-50 [18] model rather than VGG16 [19] and MobileNetV1 [20]. These authors partitioned the severity of FHB infestation into 5 classes. Zhang et al. [9] combined hyperspectral and RGB imaging data for FHB detection, also using dissected ears. In contrast, Gu et al. [21] used an ordinal scale with 5 classes for classifying FHB severity of individual dissected ears. These authors used the AlexNet [22], pretrained on ImageNet [17] for extracting deep features, which were combined with shallow color and texture features. Using a Relief-F algorithm followed by classification, they achieved improved accuracy for FHB classification. Zhang et al. [23] applied a fully connected network for ear segmentation, followed by a pulse-coupled neural network with *K*-means clustering of an improved artificial bee colony for diseased ear area segmentation. Using in situ RGB imaging in the field, Qiu et al. [24] developed a segmentation method for discriminating diseased from healthy ear pixels based on a green/blue color channel feature. They used a Mask R-CNN [25] model, pretrained on the COCO dataset [26], for the segmentation task. However, the method was developed with only a few cultivars, and the images were taken under controlled illumination. Gao et al. [27] used a tandem dual BlendMask deep learning algorithm for simultaneously segmenting ears and diseased pixel area. The authors used a feature pyramid network based on the ResNet-50 model [18]. In addition, they defined the FHB severity as the “proportion of the diseased area to the total spike area” [27]. In a similar approach, Su et al. [28] applied a Mask R-CNN based on a feature pyramid network with ResNet-101 [18] for predicting diseased FHB area per ear after wheat mask generation. Recently, Hong et al. [29] developed a lightweight YOLOv4 [30] model, adapted by the MobileNet [20] for lightweight FHB detection from unmanned aerial vehicle (UAV)-based images. The suggested model was based on the labeling of diseased areas using bounding boxes. Xiao et al. [31] used UAV-based hyperspectral data for extracting spectral and texture features, which were used for the identification and spatial mapping of lightly and heavily infested areas.

The development of sensor-based phenotyping methods relies on the quantity and quality of the reference data [6,32]. Commonly, available reference data are limited to the assessment of one expert (rater). Intrarater reliability of experienced raters is generally high [33], so that models trained and tested with data scored by the same person often appear to provide sufficient accuracies. However, interrater reliability, describing the reproducibility as compared to the assessment by another expert, is often substantially lower [32]. Thus, the transfer of sensor-based models to data assessed by other raters was rarely addressed and remains uncertain. On the other hand, the transfer of plant disease models between datasets generated in different years and growth stages was rarely addressed but is essential for practical applications, notably in the case of FHB, where the main symptom of whitened ears overlaps with senescence effects. Thus, model generalizability needs to be evaluated from the difference in model performance between the training data and the test data (unseen data) [34]. For wheat ear blast, which causes similar symptoms, Fernandez-Campos et al. [35] reported better classification accuracies for a test dataset with only premature ears than for a dataset with premature and mature ears. However, the training data comprised data from both development stages. While models must provide sufficient accuracies within individual datasets, i.e., years, it remains unclear to what extent models trained and tested on data from different years, or on combined data from multiple years, perform on unseen data.

While RGB-based FHB detection was substantially improved in the past years [15], most previous approaches either gathered images of dissected ears under controlled conditions or used a method comprising the detection of ears and the percentage of diseased pixels [23,24,27,28]. However, this approach is susceptible to FHB gradients within the images and the differing pixel size of the ears. Furthermore, improving pixel-based approaches requires time-intense image labeling. Moreover, traditional visual scoring is often based on an ordinal scoring scale without explicit estimation of the percentage of diseased spikelets or pixels [16,21]. Therefore, this study aims to develop and test a classification algorithm for directly estimating FHB scoring values without prior segmentation of overall or diseased ear pixels. In addition, we aim for a cost-effective method and deliberately use RGB images captured with consumer-grade cameras under field conditions with different lighting conditions. We use a pretrained EfficientNet [36] architecture in its smallest variant (b0) to classify the FHB severity. Recently, the EfficientNet has proven to be useful for plant disease phenotyping for its superior combination of high accuracy and calculation efficiency [37–39]. In our implementation, it takes RGB images as inputs and returns a numeric value that represents the severity of FHB. The network is faster and smaller than other networks used for FHB classification, such as ResNets or MobileNets [16], while also potentially deployable on mobile devices. We compare our model with the interrater reliability and evaluate the performance on individual years of our datasets and on unseen data, which is important for real-world applications.

## Material and Methods

### Data acquisition

#### Experimental design

The winter wheat *Fusarium* trials were conducted in 2020, 2021, and 2022 in Southeast Germany. The sowing dates were 25, 26, and 29 October 2019, 2020, and 2021, respectively. Preceding crops were rapeseed for the first and third and sugar beet for the second year. See [40] for details on soil and weather conditions. The wheat was sown in double-row plots of 1.5 m in length and with a row distance of 15 cm. The germplasm consisted of preselected material, F5 generation and older, and double haploid lines without extreme genotypes in terms of morphology and phenology. The breeding program is targeted to the central European market, very early or very short phenotypes are therefore missing. In the larger part of the trials, every fifth plot was sown with 1 of 2 reference cultivars. *F. culmorum* spores were inoculated on 2 dates each in both years as a solution of 600 liters·ha^−1^ with a concentration of 100,000 spores·liter^−1^. Inoculation dates were 4 and 8 June 2020, 15 and 18 June 2021, and 30 May and 1 June 2022, corresponding to early and late milk ripeness, respectively.

#### Annotation and image acquisition

The annotation scale for FHB is separated into 9 severity levels, ranging from severity level 1 to 9. The FHB severities follow an ascending logarithmic order [41]. The annotated scores predominantly refer to the percentage of the infested spike area as averaged over the spikes of each plot, as shown in Table 1. For example, classes 1, 5, and 8 correspond to 0%, 8% to 14%, and 37% to 61%, respectively. The averaged score is a combination of the number of infested spikes and their respective infestation severity. For example, 50% infested spikes of each 100% infested area would result in an overall infestation area of 50% and, therefore, class 8, while 10% infested spikes of each 10% infested area would result in an overall infestation area of 1% and, therefore, class 1 [41]. In addition, the intensity of the color change was considered if the area-based score was at the boundary of 2 classes.

**Table 1.** The FHB severity annotation scale. The scale is based on the official guidelines of the German Federal Office of Plant Varieties (Bundessortenamt [41], p. 2.7–3).

FHB class	Infested spike area (%)	Description
1	0	Missing
2	0–2	Very low to low
3	2–5	Low
4	5–8	Low to medium
5	8–14	Medium
6	14–22	Medium to strong
7	22–37	Strong
8	37–61	Strong to very strong
9	61–100	Very strong

The severity of FHB infestation was directly scored by a breeder in the field, which is the established method for FHB assessment. However, since image capture dates differed from those of these field annotations, image-based annotation was conducted instead on the corresponding image data. For each image, 2 raters assigned one score representing the average infestation level. RGB images of the *Fusarium* plots were captured manually using consumer-grade cameras from the front of the plots, since the plots were accessible from unvegetated cross tracks only. The cameras were positioned at breast height, and the angle was adjusted to capture the entire plot. To account for changing illumination conditions, all camera settings were set to automatic adjustment. In most cases, a wide-angle zoom was chosen. We recorded the FHB infestation and images in 3 consecutive years, 2020, 2021, and 2022. Because of the size of the trials and lacking accessibility of about half of the plots, images were captured only in a subset of the overall plots. In 2021, some plots were characterized by insufficient germination. Therefore, images of plots with less than 50% plant cover were discarded. In addition, different cameras were used for image recording. Table 2 summarizes the data collection with respect to measurement dates and camera equipment.

**Table 2.** Dataset image, annotation, and camera type information.

Years	Image capture date	Cameras	Original resolution
2020	23.06	Panasonic DMC-TZ4	3,264 × 2,448
	03.07		
2021	06.07	Panasonic DMC-TZ4	3,264 × 2,448
		NIKON 3700	2,048 × 1,536
2022	30.06	Panasonic DMC-TZ4	3,264 × 2,448
		Panasonic DC-GH5	5,184 × 3,888

**Fig. 1.**
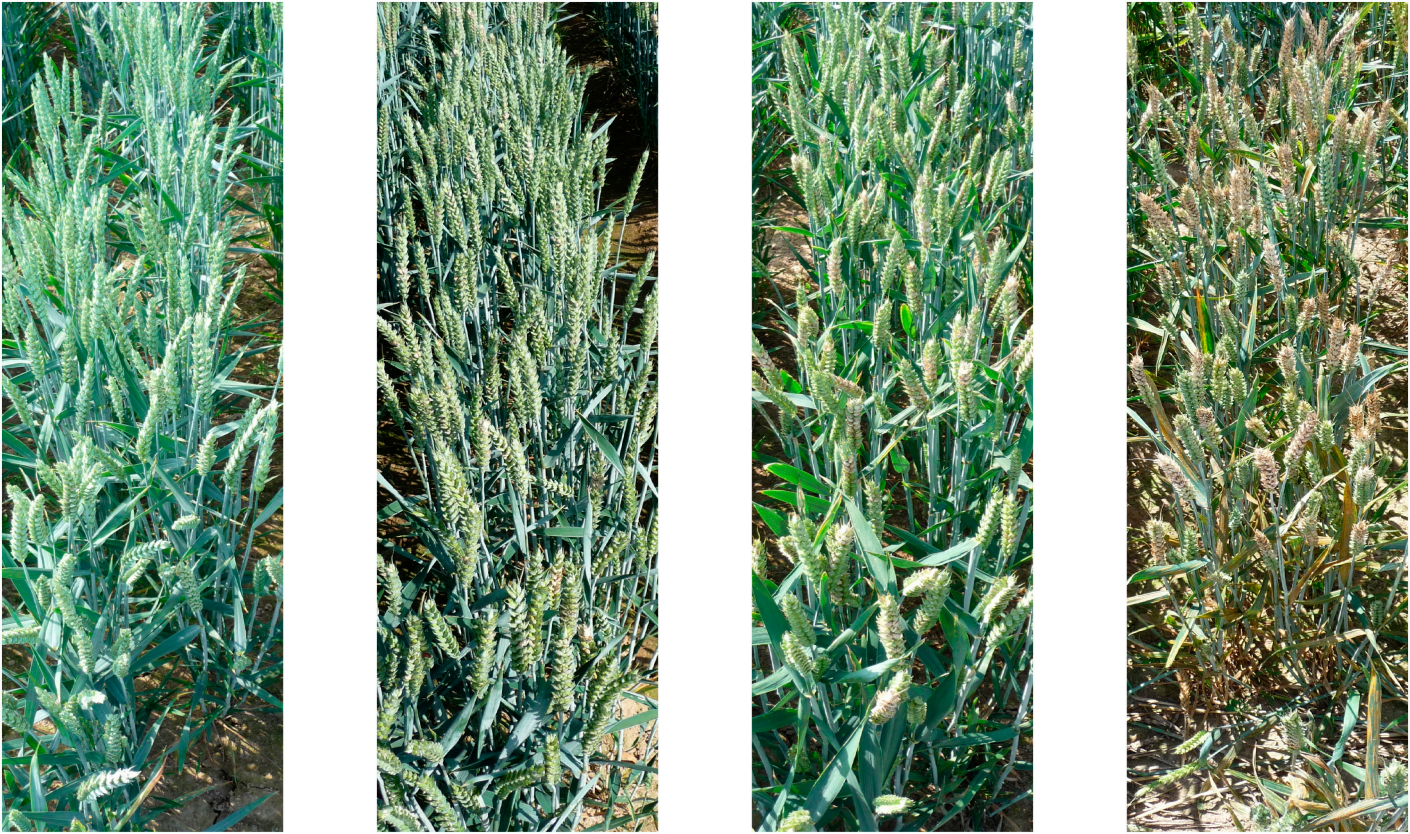
Dataset examples with an increasing FHB severity from left to right.

We designed the dataset such that each image depicts exactly one plot. The images were manually cropped using the image editing software IrfanView so that only the main plots were visible, with hardly any information about the neighboring plots. If needed, images were rotated before using rectangular cropping. Figure 1 shows examples of our dataset with an increasing FHB severity from left to right.

Rating by 2 persons results in different distributions in the annotation assignment. Table 3 shows the number of images the raters assigned to the classes, with different class distributions by raters. We use the following notations that we will also use in the subsequent sections: Dataset D refers to the data collection of a specific year, $D_{20}$ for 2020, $D_{21}$ for 2021, and $D_{22}$ for 2022. The 2 recording dates of $D_{20}$ were combined to get a larger dataset for 2020. Image rater $I_{n}$ refers to the person who performed the annotation based on the captured images with *n* as a specifc rater. The total amount of images of $I_{1}$ and $I_{2}$ differs from each other because the raters omitted images for which they were unsure about assigning an appropriate annotation or because of insufficient image quality, e.g., strong image illumination.

**Table 3.** Number of rater annotations separated into the FHB severities.

Data	Rater	FHB severity									∑
		1	2	3	4	5	6	7	8	9	
$D_{20}$	$I_{1}$	56	241	139	79	71	72	79	32	4	**773**
	$I_{2}$	36	219	233	82	85	80	47	8	1	**791**
$D_{21}$	$I_{1}$	12	121	191	240	327	334	285	79	6	**1595**
	$I_{2}$	1	47	283	481	309	198	85	4	0	**1,408**
$D_{22}$	$I_{1}$	7	60	65	68	46	69	125	74	24	**538**
	$I_{2}$	9	56	62	55	82	97	134	86	18	**595**

I, Image rater.

### Image data preprocessing

The different datasets were divided into training, validation, and test sets. We used 80% of the total dataset for the training process, of which we used 80% for training optimization and 20% for validation. The remaining 20% of the total dataset was used for testing the resulting networks. We used the same data split percentages for all years and split them separately. In addition, we performed a stratified split of the labels to obtain the original label distribution of the entire dataset in each data split. Since we also used multiple cameras for the images, we also split across each camera in a stratified manner. For example, if multiple cameras for a year of data collection were used, we keep the distribution of annotated images per camera and merge these splits to create the data splits for that data year.

**Fig. 2.**
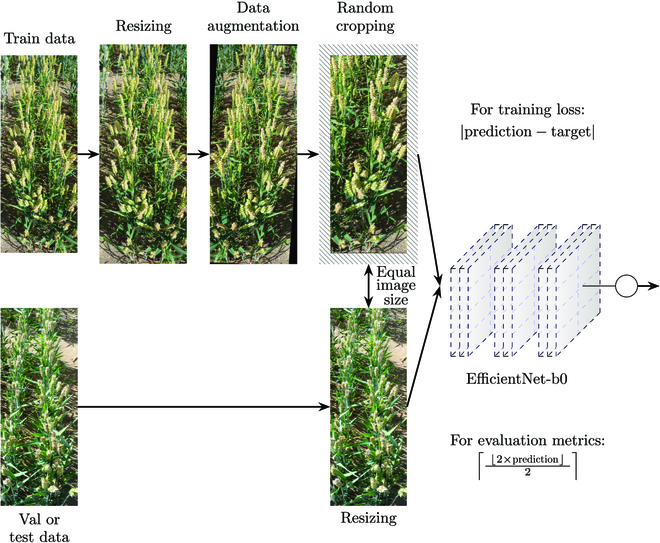
Data pipeline for the training process of the EfficientNet-b0 network and the data transformations for applying the validation and test data to the network.

Training reasonable image classification networks requires sufficiently large datasets. We used data augmentation methods to meet this requirement and to contribute to a better generalization of the network. In particular, we used resizing, random rotation with a maximum rotation angle of 2.5°, random cropping, random horizontal flipping, and image normalization. We iterated over all images in advance to calculate the average height and width for resizing and the normalization values (mean, SD) for the image color channels. Random cropping was used so that 90% of height and width must always be maintained. It was found that adjustments in color space decrease the learning potential of FHB severity with our dataset. This may be because both the color and brightness of the wheat ears are crucial for correct classification. Such augmentation in the color spectrum could lead to a modification of these indicators and, subsequently, a fluctuation in the actual FHB severity. Adjustments in the color spectrum may reduce or increase the separation of the color regions too much, resulting in ambiguous classification. For validation and testing, only resizing and data normalization were used. In addition, to obtain the input sizes for all data splits, the training augmentation methods of resizing and random cropping result in the same image height and width as the resizing for validation and test datasets. The augmentation pipeline for training, validating, and testing is illustrated in Fig. 2. To train the neural networks, we only used the annotations of either the rater $I_{1}$ orand $I_{2}$. The reason is that the network and the raters make their final decision based on the image. Thus, the classification is performed exclusively on the basis of the same underlying information.

While the requirement of the amount of data was met by data augmentation, further aspects had to be considered. In particular, only a few images and annotations are available for severities of very low and very high *Fusarium* infestation, as shown in Table 3. Even with data augmentation and the weighted random sampler, overfitting can occur for a few samples. Therefore, we combined the severities 1 and 2 as well as 7, 8, and 9, respectively. This results in 6 classes for the classification task of FHB, {≤2, 3, 4, 5, 6, ≥7}. Furthermore, we did not run a network training for the dataset $D_{22}$, as the total amount of images would be too small for a meaningful prediction and evaluation.

### The EfficientNet regression approach

To classify the images according to the severity of FHB, we used a state-of-the-art neural network for image classification. Specifically, we used the EfficientNet [36] (size b0) with pretrained weights on the ImageNet dataset [17]. However, deeper models did not show higher performance on our dataset and only resulted in a longer processing time (data not shown).

To enable the application on our dataset, we replaced the output layer of the pretrained network. A network output layer that uses the number of specified FHB severities as the number of output neurons was used. We freeze all pretrained weights during the first 2 epochs and only train the new classification output layer. After the first 2 epochs, we unfreeze all weights and train the network for a maximum of 50 epochs. The freezing method was used to adapt the network to the new output layer and to avoid losing previously learned information due to high gradients at the beginning of the training. Early stopping was used with a loss delta of 1 × 10^−2^ and a patience of 10 epochs. We set the learning rate to 1 × 10^−4^ and the batch size to 4 and used Adam [42] for stochastic weight optimization.

Since the annotations are subject to an ordinal scale, both a classification and a regression task can be performed [43]. In the classification variant, the neural network has as many output neurons as classes and uses the index of the neuron with the highest output value as the prediction value. The regression approach, on the other hand, uses only one output neuron and rounds its output value to an integer value to assign a unique class. We found slightly better results for the regression model (not shown), which was implemented with the L1 loss. Therefore, the results presented in the following sections are always based on the EfficientNet regression network. The L1 loss reduced the discrepancies because mispredictions with high deviations from the actual annotation are penalized stronger than with the categorical cross-entropy loss.

The number of images per FHB severity plays a crucial role in training the neural network. That is because the loss of the model can be minimized by focusing on the most represented annotation label only. Since we have very diverse amounts of images for the different FHB severities, we have to use additional methods to train the model.

To reduce specialization on a particular label and reduce overfitting, we found that using a weighted random sampling method helps to generalize the network for our dataset. The weighted random sampler helps to sample images of different labels so that the number of images of each severity is represented equally during a training epoch. For the sampling process, the sampling weight *w* of each image sample $s_{l}^{i}$ of the dataset with label *l* ∈ *L* is calculated by $w^{s_{l}^{i}}=\frac{|l|}{|L|}$ with *L* = {1, 2, …, 9} as the set of labels, ∣*L*∣ as the cardinality of *L*, ∣*l*∣ as the number of images of label *l*, and *i* as the index of a sample of *l*. Given the calculated fixed weight (probability) for each sample $s_{l}^{i}$, we use a multinomial distribution to sample images from the dataset. Therefore, images categorized into a severity with a small number of image representations are sampled more frequently than images of labels with a higher number of image representations.

Hereafter, the notation $N_{I_{n}}$ is used for neural networks trained with the training data of the rater $I_{n}$. We implemented the source code with Python and trained the networks with PyTorch and an NVIDIA RTX A6000 graphics card. Training a single network took less than an hour on average. Models were trained within the year-specific dataset separated for the annotation data of each rater. This results in 4 network trainings with the data of $I_{1}$ and $I_{2}$ for both datasets $D_{20}$ and $D_{21}$. In comparison, averaging the annotations of both raters was tested, but this approach led to markedly worse results. Because of the insufficient number of data points, the dataset $D_{22}$ was not used for model training but only for testing. Since 2 reference cultivars were grown more frequently in the trials, predictions were also compared for the plots of each of these cultivars as compared to the other plots.

### Evaluation metrics

We have 2 sources of annotations per image available for our dataset and used Cohen’s kappa *κ* metric [44] for our evaluation purposes. Cohen’s kappa is a statistical measure to assess the interrater reliability of 2 raters and is thus a measure of objectivity and is defined as follows:

$$\kappa =\frac{p_{o}-p_{e}}{1-p_{e}}$$ (1)

with *p_o_* as the relative raters’ agreement and *p_e_* as the proportional expected by chance agreement. Cohen’s kappa ranges from −1 to 1, where *κ* = 1 indicates complete agreement among raters and *κ* ≤ 0 indicates no agreement or a by-chance rating. By default, the metric assigns the same value to all disagreements. However, our dataset annotations are based on an ordinal scale. Cohen’s kappa must be adjusted to assess the extent of disagreement according to the distance of the mispredictions. Strong disagreements should have a more significant negative impact on *κ* than minor disagreements. We, therefore, used a linear weighting for the disagreements as proposed by Cicchetti and Allison [45]. When mentioning *κ* in the following, we always refer to Cohen’s kappa with linear weighting. We used only the test data split to compute all interrater *κ* values to ensure comparability with the neural network results and evaluations.

Furthermore, we used the accuracy for our evaluations, which is defined as follows [46]:

$$\text{Accuracy}=\frac{\text{correctly identified samples}}{\mathrm{all}\;\text{samples}}$$ (2)

Following the regression approach described in the “The EfficientNet regression approach” section, we round up the neural network outputs for decimals of ≥0.5 and round down for decimals of <0.5, calculated as

$$\text{Output}=\left\lceil \frac{\left\lfloor 2\times \text{prediction}\right\rfloor }{2}\right\rceil$$ (3)

for evaluation purposes. In addition, we clip the network outputs at the boundaries of the severity annotations for evaluation so that all network outputs are within the FHB severity annotation range. While we used the accuracy metric as an overall performance metric, we used precision *p* and recall *r* to evaluate individual labels, which are defined as follows:

$$\text{Precision}\;p=\frac{\mathrm{TP}}{\mathrm{TP}+\mathrm{FP}}$$ (4)

$$\text{Recall}\;r=\frac{\mathrm{TP}}{\mathrm{TP}+\mathrm{FN}}$$ (5)

with TP as the true positives, FP as the false positives, and FN as the false negatives. The precision determines the correctly predicted positive samples compared to all positive predictions. The recall determines the correctly predicted positive samples compared to all positive references.

We also used the *F*_1_ score, which calculates the harmonic mean of the 2 metrics, precision *p* and recall *r*. The formula to calculate the *F*_1_ score*_l_* individually for each label *l* is defined as follows:

$$F_{1}\;\text{scor}e_{l}=2\times \frac{p_{l}\times r_{l}}{p_{l}+r_{l}}.$$ (6)

Since we have multiple labels with a varying number of instances per label, we used the weighted $F_{1}^{w}$ score, which is calculated as follows:

$$F_{1}^{w}\;\text{score}=\sum_{l}^{L}f_{l}^{w}\times F_{1}\;{\text{score}}_{l}$$ (7)

with $f_{l}^{w}=\frac{|l|}{\sum_{l}^{L}‍|l|}$, *L* as the set of labels, and ∣*l*∣ as the number of instances of *l* ∈ *L*.

To assess the quality of the regression models without rounding the output to the nearest integer, we used the root mean squared error (RMSE), which is defined as follows [47]:

$$\mathrm{RMS}E_{l}=\sqrt{\frac{\sum_{i\in l}^{|l|}‍{\left({\text{prediction}}_{i}-{\text{target}}_{i}\right)}^{2}}{|l|}}$$ (8)

## Results

### Interrater reliability

**Fig. 3.**
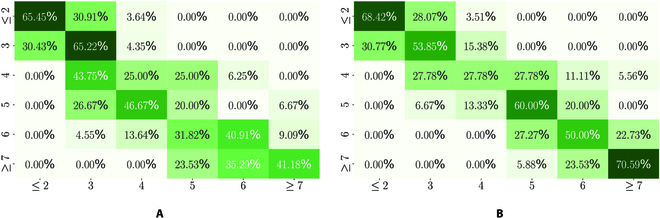
Confusion matrix of $I_{1}$ versus $I_{2}$ (A) and $I_{1}$ versus $N_{I_{1}}$ (B), both from data $D_{20}$. *y* axis is $I_{1}$. The confusion matrices for all other comparisons by years, raters, and networks can be found in Figs. 1 to 4.

Before evaluating the network results, the raters’ matches or mismatches are evaluated using Cohen’s kappa *κ*. The interrater reliability of the raters is shown in Table 5 (last column). Cohen’s kappa shows that the image raters $I_{1}$ and $I_{2}$ depict moderate equality. Furthermore, data from the year 2021 also show higher disagreement between the image raters than in 2020. The agreement of the raters $I_{1}$ and $I_{2}$ is shown in the confusion matrix in Fig. 3A. It is noticeable that the deviation from the image raters is usually no more than one severity level. The *Fusarium* scores differed substantially between scoring dates and are related to the genotypic differences in resistance and the 2 inoculation dates.

### Neural network performance

All results refer to the fully trained neural network and subsequent evaluation using only the test datasets, if not stated otherwise.

#### Network-to-rater reliability

We again used Cohen’s kappa *κ* to compare the trained networks with the annotation agreement of the raters, shown in Table 5. We follow the notation style from the “Interrater reliability” section and use the notation $N_{I_{n}}$ to refer to a trained neural network with data annotations from rater $I_{n}$. Compared to the agreement of the raters from Table 5, the agreements between the respective rater and the specifically trained neural network increase, as shown in Table 5. This implies that the respective networks succeed in adapting to the specific assessments of the raters. In addition, the reduction of disagreement for $D_{20}$ is illustrated with the confusion matrices, shown in Fig. 3B. Figure 3A shows the annotation comparison between the raters $I_{1}$ and $I_{2}$. Figure 3B shows the comparison of the rater $I_{1}$ and the network $N_{I_{1}}$. The spread on the main diagonal decreases with the neural network approach, resulting in a lower deviation of the predictions from the actual targets. This pattern also appears for $D_{21}$ and for networks trained on annotations of rater $I_{2}$ (see Figs. S1 to S4).

#### FHB severity evaluation: The rater effect

To measure the exact match of the raters and the network predictions, we used the metrics accuracy, weighted $F_{1}^{w}$ score ($F_{1}^{w}$), precision (*p*), and recall (*r*). A specific overview of the severity prediction results is presented in Table 4.

**Table 4.** Rater and network precision *p*, recall *r*, *F*_1_*^w^* score and accuracy (Acc) evaluation results.

Comparison	Data	FHB severity												$F_{1}^{w}$	Acc
		≤2		3		4		5		6		≥7			
		*p*	*r*	*p*	*r*	*p*	*r*	*p*	*r*	*p*	*r*	*p*	*r*		
$I_{1}\;\mathrm{versus}\;I_{2}$	$D_{20}$	0.84	0.65	0.34	0.65	0.24	0.25	0.17	0.20	0.56	0.41	0.70	0.41	0.52	0.50
	$D_{21}$	0.75	0.29	0.28	0.50	0.26	0.54	0.32	0.33	0.36	0.27	0.73	0.17	0.32	0.33
$I_{1}\;\mathrm{versus}\;N_{I_{1}}$	$D_{20}$	0.83	0.68	0.39	0.54	0.38	0.28	0.43	0.60	0.55	0.50	0.67	0.71	0.59	0.57
	$D_{21}$	0.69	0.52	0.34	0.36	0.36	0.43	0.45	0.43	0.38	0.49	0.80	0.53	0.48	0.47
$I_{1}\;\mathrm{versus}\;N_{I_{2}}$	$D_{20}$	0.57	0.89	0.66	0.40	0.75	0.55	0.14	0.43	0.56	0.48	0.00	0.00	0.53	0.53
	$D_{21}$	0.50	0.54	0.56	0.51	0.53	0.51	0.49	0.51	0.52	0.59	0.50	0.18	0.50	0.50

The neural networks achieve better results compared to the rater matches ($I_{1}$ versus $I_{2}$), for both trained neural networks $N_{I_{1}}$ and $N_{I_{2}}$. Furthermore, the network trained with the data annotations of $I_{1}$ is superior to the accordance of the 2 raters $I_{1}$ versus $I_{2}$ in all respects, as visible from the weighted $F_{1}^{w}$ score and the overall accuracy, for both test datasets of $D_{20}$ and $D_{21}$.

To fully utilize the numeric values generated by the regression model, we applied the RMSE to assess the deviation (distance) of the predictions from the actual targets, shown in Table 5. The comparison between the raters and the networks trained with the annotations from the individual raters shows that the trained networks reduce the prediction errors as compared to the interrater deviation. That aligns with the results of the visualization of the confusion matrices in Fig. 3, resulting in fewer large label mispredictions, i.e., deviations of the predictions and targets of more than one FHB severity. Therefore, the performance of our network is comparable or superior to that of human raters.

**Table 5.** Rater and network RMSE and *κ* evaluation results.

Comparison	Data	FHB severity						RMSE_all_	*κ*
		≤2	3	4	5	6	≥7		
		RMSE	RMSE	RMSE	RMSE	RMSE	RMSE		
$I_{1}$ versus $I_{2}$	$D_{20}$	0.67	0.59	0.97	1.34	1.17	1.14	**0.93**	**0.68**
	$D_{21}$	1.23	1.13	0.81	1.0	1.57	1.74	**1.34**	**0.40**
$I_{1}$ versus $N_{I_{1}}$	$D_{20}$	0.65	0.68	1.22	0.77	0.71	0.69	**0.77**	**0.76**
	$D_{21}$	0.87	1.07	1.12	0.88	0.82	0.81	**0.91**	**0.62**
$I_{1}$ versus $N_{I_{2}}$	$D_{20}$	0.33	0.77	0.67	1.0	0.82	1.48	**0.75**	**0.70**
	$D_{21}$	1.07	0.84	0.75	0.76	0.85	0.91	**0.81**	**0.59**

#### FHB severity evaluation: The dataset effect

Previous FHB network training reported in the literature was predominantly evaluated on the data from the same trial. In the following, we show (a) the results for trained networks with 1 year and tested on another year and (b) the results for trained networks with 2 data years and tested on a single data year. All networks were trained with the data annotations from $I_{1}$ since the annotations provided better results for the severity evaluation compared to $I_{2}$. Accordingly, we also used the test datasets of $I_{1}$ for the network evaluation. We also used $D_{22}$ entirely (no train/val/test splits) to evaluate the generalization of the networks. For the datasets $D_{20}$ and $D_{21}$, we only used the test datasets so that the results are comparable with the evaluations from the “FHB severity evaluation: The rater effect” section. Tables 6 and 7 show all results of the dataset effect evaluation for the networks trained on $D_{20}$, $D_{21}$, and $D_{20+21}$. Starting with the trained networks $N_{1}$, from the “FHB severity evaluation: The rater effect” section, for both years 2020 and 2021 individually, we cross-test the networks with the datasets from different years. Training on $D_{20}$ and testing on $D_{21}$ perform slightly better than the interrater comparison $I_{1}$ and $I_{2}$ in terms of $F_{1}^{w}$ and accuracy (Table 4) but worse when compared with training exclusively on $D_{21}$. Comparing the results with the RMSE and *κ* results, the networks perform slightly worse, indicating that the network also predicts higher deviations for some data points. In contrast, training on $D_{21}$ and testing on $D_{20}$ perform slightly worse than the interrater comparison $I_{1}$and $I_{2}$ in terms of $F_{1}^{w}$ and accuracy but slightly better when evaluated by the RMSE and *κ*. The reason for the lower RMSE_all_ is that the network predicts the severity levels with major occurrences, e.g., severity level of ≤2, better compared to the deviations of the raters. Testing on $D_{22}$ results in low $F_{1}^{w}$*κ* and high RMSE_all_ values for both training data years 2020 and 2021.

**Table 6.** Network performance on unseen data years and combined data years evaluated on the metrics precision *p*, recall *r*, $F_{1}^{w}$, and accuracy.

Train data	Test data	FHB severity												$F_{1}^{w}$	Acc
		≤2		3		4		5		6		≥7			
		*p*	*r*	*p*	*r*	*p*	*r*	*p*	*r*	*p*	*r*	*p*	*r*		
$D_{20}$	$D_{21}$	0.30	0.38	0.33	0.30	0.9	0.45	0.32	0.45	0.32	0.44	0.82	0.12	**0.33**	**0.34**
$D_{20}$	$D_{22}$	0.30	0.34	0.23	0.37	0.25	0.28	0.26	0.43	0.20	0.48	0.95	0.17	**0.29**	**0.29**
$D_{21}$	$D_{20}$	0.67	0.84	0.23	0.19	0.36	0.28	0.21	0.27	0.36	0.36	0.83	0.29	**0.47**	**0.48**
$D_{21}$	$D_{22}$	0.92	0.18	0.33	0.32	0.18	0.23	0.14	0.41	0.18	0.49	0.82	0.13	**0.25**	**0.25**
$D_{20+21}$	$D_{20}$	0.80	0.84	0.52	0.46	0.31	0.28	0.41	0.47	0.58	0.64	0.67	0.59	**0.61**	**0.62**
$D_{20+21}$	$D_{21}$	0.86	0.57	0.40	0.52	0.32	0.36	0.46	0.43	0.39	0.60	0.87	0.34	**0.49**	**0.46**
$D_{20+21}$	$D_{22}$	0.94	0.22	0.33	0.29	0.38	0.29	0.14	0.28	0.17	0.36	0.72	0.55	**0.43**	**0.40**

**Table 7.** Network performance on unseen data years and combined data years evaluated on the metrics RMSE and Cohen’s kappa (*κ*).

Train data	Test data	FHB severity						RMSE_all_	*κ*
		≤2	3	4	5	6	≥7		
		RMSE	RMSE	RMSE	RMSE	RMSE	RMSE		
$D_{20}$	$D_{21}$	1.25	1.50	1.09	1.05	1.26	2.00	**1.44**	**0.38**
$D_{20}$	$D_{22}$	1.03	0.99	1.36	1.18	1.96	2.19	**1.75**	**0.41**
$D_{21}$	$D_{20}$	0.40	0.90	1.31	1.32	1.02	0.94	**0.90**	**0.68**
$D_{21}$	$D_{22}$	1.57	1.37	1.31	1.02	1.08	1.65	**1.46**	**0.38**
$D_{20+21}$	$D_{20}$	0.58	0.79	1.25	0.97	0.60	0.54	**0.76**	**0.78**
$D_{20+21}$	$D_{21}$	0.95	1.19	1.16	0.89	0.79	0.90	**0.96**	**0.60**
$D_{20+21}$	$D_{22}$	1.61	1.59	1.66	1.08	0.89	0.97	**1.25**	**0.53**

Training a new multiyear data network on the training data of the data years 2020 and 2021 outperforms the single-year trained neural networks in almost all respects. Testing the multiyear data network on the test data of $D_{20}$ , i.e. the dataset from 2020, gives better results than the network trained on $D_{20}$. The multiyear data network also produces comparable results on the test dataset of $D_{21}$ compared to the network trained on $D_{21}$. The test results on $D_{22}$of the multiyear data network highly increase the classification metrics $F_{1}^{w}$ scores and the accuracy and reduce the regression metrics RMSE_all_ and *κ* compared to the networks trained on the data from a single year. In contrast to the years’ effect, the difference in the prediction errors between the cultivar groups was relatively small (see Table S1).

## Discussion

Nondestructive high-throughput disease phenotyping under field conditions is challenging because of (a) suboptimal data acquisition conditions, (b) the need for affordable, easy-to-use sensors, and (c) limited accessibility of the individual ears [14,15]. Therefore, deep learning algorithms have to be optimized for overcoming limitations in the image data. However, these algorithms require a large amount of sensor and scoring data [48]. The present study deliberately used in situ RGB image data to directly predict one FHB score per image. This approach resulted in lower accuracies than in a number of previous studies [9,21,24,27] but offers the advantage of being nondestructive, not requiring specific sensor setups, time-consuming object or pixel-based image annotation or image segmentation.

### The influence of the raters

Most previous FHB models did not assess the influence of multiple raters. Our results confirm that despite a predefined scoring scale, multiple raters differ substantially with respect to their agreement of the FHB severity level, notably in $D_{21}$. This is in line with several studies on the comparison of interrater reliability as reviewed in [32] and likely influenced by the heterogeneous nature of the field-based images. Bock et al. [32] reported a tendency to prefer values by individual raters, higher intrarater reliability with increasing rating experience, and a nonlinear bias in the rated values as error sources. In the present dataset, rater $I_{1}$ tended to exploit the scoring scale more toward both severity extremes, which was evident, especially in $D_{21}$, the dataset with the lowest interrater reliability. Moreover, a rating can be affected by the differing estimation of the number of diseased spots and the spots’ area, which are both implicitly considered for FHB scoring. Tentatively using the averaged scoring data from both raters decreased the network performance (data not shown). Thus, the results indicate that rater-specific models are more useful for a limited amount of data. However, for training reliable multirater models, it appears that substantially more data would be required, involving more training of the raters for achieving higher interrater reliability before the training.

### Applicability of the EfficientNet regression approach

The assessment of FHB severities is typically considered a classification task. Nevertheless, because of the ordinal scale, it is also possible to train a regression task [43], which performed better in the present study. For the classification task, we used the categorical cross-entropy loss. For the regression task, we used the L1 loss. We found that the L1 loss regression method performed slightly better than the cross-entropy loss classification method. The L1 loss reduced the discrepancies because mispredictions with high deviations from the actual annotation are penalized stronger than with the categorical cross-entropy loss. The network may perform better because the loss function used during training prioritizes minimizing the distance between the predicted and actual labels. Traditional classification methods do not take into account the ordinal nature of the labeling scale, resulting in an equivalent penalty for misclassification regardless of the degree of discrepancy between predicted and actual labels.

The assessments were conducted similarly to other studies, thus using metrics for classification tasks. That involves rounding the results of the regression model up and down accordingly to get one specific classification output. Afterward, the result can be used for the metrics precision, recall, *F*_1_ score*_l_*, and accuracy. Nevertheless, the regression model offers broader possibilities for additional evaluations due to its output in floating point precision. Furthermore, using the lightweight EfficientNet architecture allows easier deployability to mobile devices compared to previous approaches [16]. The authors of EfficientNet have introduced methods for scaling convolutional networks that not only add more layers to an existing network to achieve better results but instead to find a balance between depth, width, and resolution. They developed much smaller and faster networks with better predictive performance compared to existing convolutional network designs. We additionally used pretrained ResNet architectures of different sizes, as Gao et al. [16] have suggested for classifying FHB severities. However, these have always performed worse than the EfficientNet models in terms of processing time and prediction performance. This is in line with the highest accuracy of an adapted EfficientNet as compared to a number of other networks for apple [49] and for strawberry [39] disease detection.

### Prediction accuracy

The higher the interrater reliability of a particular year of data, the better the network performance trained on individual raters as shown in Table 5. This indicates that the networks benefited from higher scoring quality in $D_{20}$ compared to $D_{21}$. Although the networks were trained separately with individual raters, the results were better when the interrater reliability was also higher for a particular data year, as shown in Table 5. This indicates that the networks profited from higher-scoring quality in $D_{20}$ compared to $D_{21}$. In contrast, also higher soil fraction in many images in $D_{21}$ may have negatively affected the networks. This is in line with generally improving plant disease classification for various crops after the removal of soil pixels [48]. In the present study, soil pixels were not removed, however, since it can also be error-prone because of sun or shadow gradients. Compared to the observed interrater reliability, the network-to-rater reliability was higher for both raters and in both years, indicating that the network performance is better compared to the error of the reference method when ignoring rater effects. Still, the model results are weaker than in most previous studies, which, however, generally involved higher efforts for increasing image quality with respect to constant illumination conditions, camera quality, or the dissection of ears. Moreover, most previous approaches used pixel-based or object-based approaches, requiring substantially more annotation efforts. However, these requirements are important hurdles for the automated, high-throughput FHB detection under field conditions. From a practical point of view, our approach maintains still sufficient discrimination. In fact, the deviations between the predictions and the actual labels of our trained models are less dispersed across the ordinal FHB severity scale, generally deviating by no more than one label. Thus, our networks are more precise than the interrater agreement. Moreover, the precise determination of the 6 to 9 classes is often not required, while often 3 classes would be sufficient. In contrast, because of the dynamic development of FHB over time and the delayed symptoms of phenologically delayed genotypes, the evaluation of the genotypes for selection would profit more from more frequent measurements, which is enabled by an automated, sensor-based approach. In most test datasets, the RMSE for the models and between raters tended to be lower within the reference cultivars than for the other genotypes, possibly indicating that the reference cultivars profited from their more frequent occurrence in the dataset (see Table S1).

### The effect of dataset size

Many images are usually required to achieve high accuracies on image data with neural networks [34,48]. Otherwise, networks tend to overfit or lack generalization. As class imbalance can also result in poor generalization [38], we combined the classes 1 and 2 and classes 7, 8, and 9 into 2 separate categories, respectively, since we had very few samples for training the network with these classes, resulting in overall 6 classes {≤2, 3, 4, 5, 6, ≥7} for the FHB severity classification. We also used data augmentation methods to increase our training dataset and further boost the network generalization. However, we applied data augmentation methods only very carefully and omitted image color space augmentation, since the bright color of FHB is essential to classify the correct severity and infestation level. Unlike Gao et al. [16], we did not use color space adjustments such as saturation and contrast. Thus, some color transformations were previously shown to have negative effects on disease detection [50]. Nevertheless, further investigation can be conducted to analyze the effect of varying illumination conditions on the network performance, especially for very low and very high severity levels of FHB. The effect in dataset size was noticeable when training the networks with the combined datasets $D_{20}$ and $D_{21}$, as displayed in Tables 4 and 6. Compared to single-year dataset networks, we achieved better classification and regression metric scores in the multiyear dataset networks when testing on the test split of $D_{20}$ and comparable results when testing on the test split of $D_{21}$, respectively. As tested on $D_{20}$, we increased the $F_{1}^{w}$ scores by 0.02, the accuracy by 5%, and *κ* by 0.02 and decreased the RMSE_all_ by 0.01, whereas on $D_{21}$, the $F_{1}^{w}$ scores improved by 0.01, the accuracy dropped by 1% , *κ* dropped by 0.02 and the RMSE_all_ increased by 0.05. To further improve future models for FHB based on RGB image data, our dataset could be used for pretraining models.

### Transferability: The effect on unseen data from different years

To verify the applicability in real-world applications, we tested and evaluated our networks on unseen data from different years, differing substantially in phenological development, plant density, genotypic composition, and soil/plant pixel ratios.

As displayed in Table 6, the $F_{1}^{w}$ scores and accuracies generally decreased when the years of data for training and testing a model differed. This indicates a shift in the distribution of data between years of data collection that cannot be interpreted sufficiently by the model, due to the aforementioned year-specific factors. Nevertheless, as stated in the “The effect of dataset size” section, the higher the data amount, the better the model results. This is also the case for the transferability and the learning of broader data distribution, meaning that it is possible to achieve better results by collecting more diverse data from different years.

When training a model with the combined datasets $D_{20}$ and $D_{21}$, we always achieved better results on the unseen data of $D_{22}$ compared to networks trained on single-year datasets. In fact, e.g., for $D_{22}$, we increased the $F_{1}^{w}$ scores by 0.14 , the accuracy by 11%, and *κ* by 0.12 and reduced the RMSE by 475.5 compared to the best network trained on the single-year dataset $D_{20}$.

As an alternative, to further improve the variability and balance of the dataset without collecting new datasets and without requiring expert knowledge in FHB assessment, generative approaches to create synthetic image data [51] can be applied [38].

### Outlook

We developed models for field-based FHB assessment under conditions of minimum time and sensor resources for image data acquisition and image annotation. We evaluated the influence of 2 different raters, the interrater reliability, the influence of 2 different years for model training and the difference of within-years compared to across-years models. We have shown that the amount, rating quality, and similarity between training and test data are crucial for better generalization of the networks. Therefore, the models could be extended and improved with existing or new datasets annotated in a similar way. In particular, the coverage of FHB severities at the extremes of the ordinal scale has an essential impact on extending our models. By having more assessment at the severity extremes, the full scale ranging from 1 to 9 could be utilized instead of the reduction to {≤2, 3, 4, 5, 6, ≥7} as used for this work. While the developed model was an efficient highthroughput approach for nondestructive FHB estimation, the manual image cropping before the usage for the models was still time-consuming. However, this preprocessing could be easily avoided through increased space between plots, which would allow for automated plant segmentation, thus allowing for image acquisition only in plot centers, or image acquisition above the plots by UAVs or field robots.

Future work should consider implementing the models as a mobile application, since we have used a fast and mobile-sized neural network implementation (EfficientNet-b0 [36]). Moreover, the models should be transferred to drone-based data to allow for high-throughput screening of large field trials.

## Acknowledgments

We gratefully acknowledge support with image acquisition and FHB scoring by S. Oswald. **Funding:** This research was partly supported by funds of the Federal Ministry of Food and Agriculture (BMEL) based on a decision of the Parliament of the Federal Republic of Germany via the Federal Office for Agriculture and Food (BLE) under the innovation support program for the project 2818407A18. **Author contributions:** D.R. and L.P. wrote the manuscript. L.R. and A.H. conceived and designed the experiments. L.R. conducted the experiments. L.P. performed the data acquisition and data quality check. D.R. and T.S. designed the perception stack. D.R. implemented the perception stack. T.S., P.O.N., and D.C. revised the manuscript. All authors read and approved the final manuscript. **Competing interests:** The authors declare that they have no competing interests.

## Data Availability

The data used in this paper are publicly available. The image data were collected under various illumination conditions that should be used to evaluate the effects of illumination on the predictive ability of the networks, taking into account the potentially disruptive effect of direct sunlight. We provide all dataset recordings, separated into a camera-specific structure. All images and corresponding annotations can be downloaded from the link provided in our GitHub repository: https://github.com/cvims/FHB_classification.

## Supplementary Materials

Supplementary 1 Figure S1. Confusion matrix of $I_{2}$ versus $N_{I_{2}}$ , data $D_{20}$, and *y* axis is $I_{2}$.
Figure S2. Confusion matrix of $I_{1}$ versus $I_{2}$, data $D_{21}$, and *y* axis is $I_{1}$.
Figure S3. Confusion matrix of $I_{1}$ versus $N_{I_{1}}$, data $D_{21}$, and *y* axis is $I_{1}$.
Figure S4. Confusion matrix of $I_{2}$ versus $N_{I_{2}}$ , data $D_{21}$, and *y* axis is $I_{2}$.
Table S1. Comparison of reference cultivars informer, Bosporus, and all others (as one group).
