# Supplementary material for: Efficient Noninvasive FHB Estimation using RGB Images from a Novel Multiyear, Multirater Dataset
Source: Plant Phenomics. 2023 Jul 14;5:0068. doi: 10.34133/plantphenomics.0068 (PMC10348660; doi:10.34133/plantphenomics.0068)
Supplement: Supplementary 1 — Figure S1. Confusion matrix of I2 versus NI2 , data D20, and y axis is I2. Figure S2. Confusion matrix of I1 versus I2, data D21, and y axis is I1. Figure S3. Confusion matrix of I1 versus NI1, data D21, and y axis is I1. Figure S4. Confusion matrix of I2 versus NI2 , data D21, and y axis is I2. Table S1. Comparison of reference cultivars informer, Bosporus, and all others (as one group). [file plantphenomics.0068.f1.pdf]

Supplementary Materials

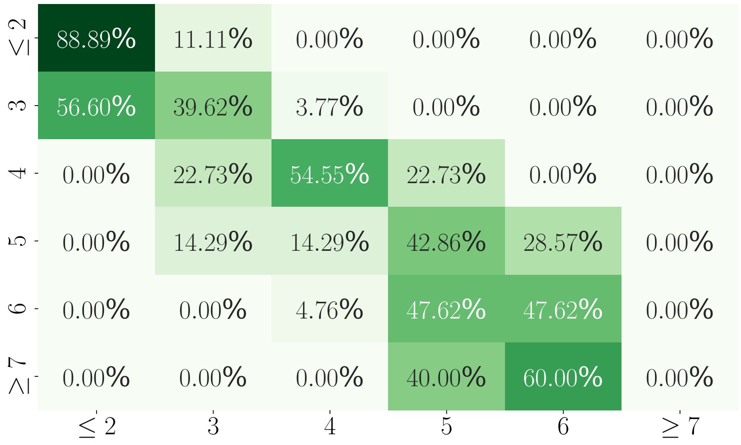

Supplementary Figure 1: Confusion matrix of $\mathcal{I}_2$ vs. $\mathcal{N}_{\mathcal{I}_2}$, Data $\mathcal{D}_{20}$, Y-axis is $\mathcal{I}_2$.

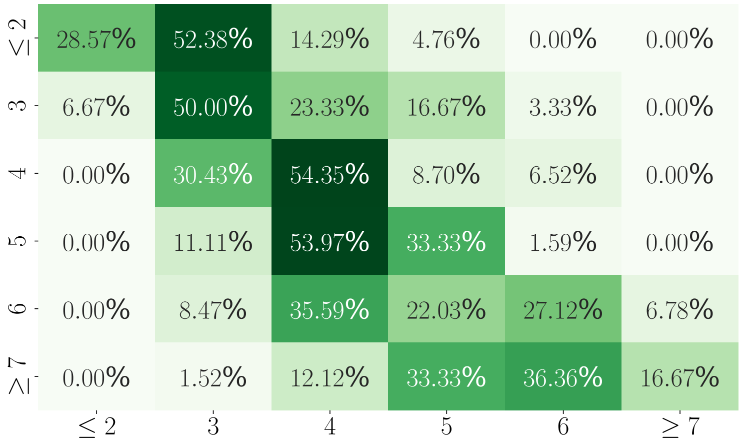

Supplementary Figure 2: Confusion matrix of $\mathcal{I}_1$ vs. $\mathcal{I}_2$, Data $\mathcal{D}_{21}$, Y-axis is $\mathcal{I}_1$.

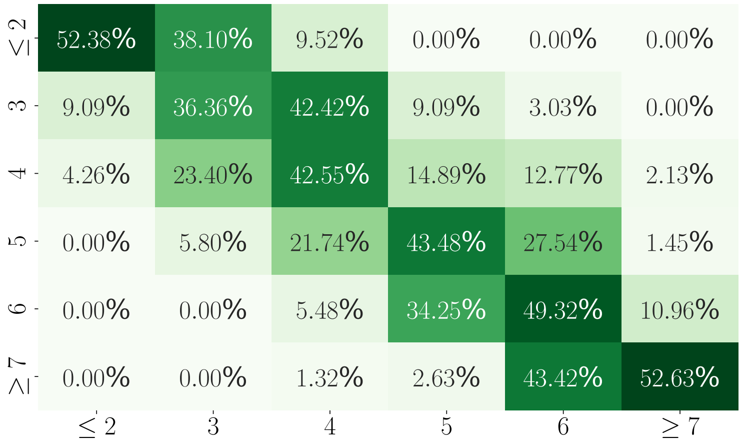

Supplementary Figure 3: Confusion matrix of $\mathcal{I}_1$ vs. $\mathcal{N}_{\mathcal{I}_1}$, Data $\mathcal{D}_{21}$, Y-axis is $\mathcal{I}_1$.

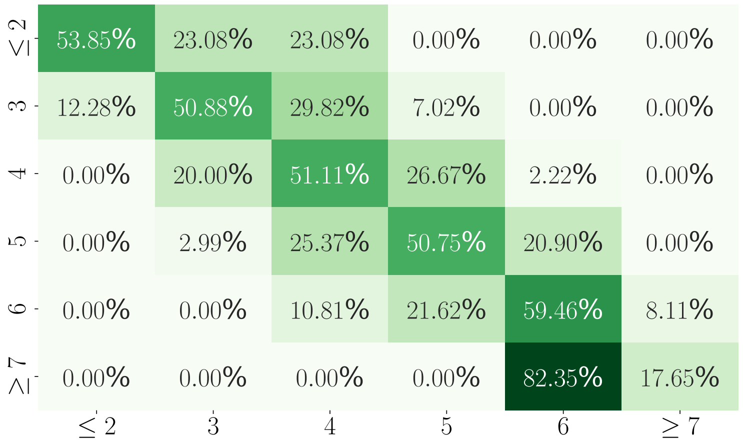

Supplementary Figure 4: Confusion matrix of $\mathcal{I}_2$ vs. $\mathcal{N}_{\mathcal{I}_2}$, Data $\mathcal{D}_{21}$, Y-axis is $\mathcal{I}_2$.

Supplementary Table 1: Comparison of reference cultivars Informer, Bosporus and all others (as one group). RMSE refers to the Root Mean Squared Error and N refers to the number of samples used for the evaluation of the FHB severity.

**Comp**	**Type**	**Data**	RMSE	N
$\mathcal{I}_1$ vs. $\mathcal{I}_2$	Informer	$\mathcal{D}_{20}$	**1.34**	4
		$\mathcal{D}_{21}$	**1.79**	25
$\mathcal{I}_1$ vs. $\mathcal{I}_2$	Bosporus	$\mathcal{D}_{20}$	**.71**	6
		$\mathcal{D}_{21}$	**1.54**	14
$\mathcal{I}_1$ vs. $\mathcal{I}_2$	Others	$\mathcal{D}_{20}$	**.95**	134
		$\mathcal{D}_{21}$	**1.24**	241
$\mathcal{I}_1$ vs. $\mathcal{N}_{\mathcal{I}_1}$	Informer	$\mathcal{D}_{20}$	**.63**	4
		$\mathcal{D}_{21}$	**.99**	25
$\mathcal{I}_1$ vs. $\mathcal{N}_{\mathcal{I}_1}$	Bosporus	$\mathcal{D}_{20}$	**.30**	6
		$\mathcal{D}_{21}$	**.67**	14
$\mathcal{I}_1$ vs. $\mathcal{N}_{\mathcal{I}_1}$	Others	$\mathcal{D}_{20}$	**.75**	134
		$\mathcal{D}_{21}$	**.92**	241
$\mathcal{I}_2$ vs. $\mathcal{N}_{\mathcal{I}_2}$	Informer	$\mathcal{D}_{20}$	**.50**	4
		$\mathcal{D}_{21}$	**.75**	25
$\mathcal{I}_2$ vs. $\mathcal{N}_{\mathcal{I}_2}$	Bosporus	$\mathcal{D}_{20}$	**.58**	6
		$\mathcal{D}_{21}$	**.93**	14
$\mathcal{I}_2$ vs. $\mathcal{N}_{\mathcal{I}_2}$	Others	$\mathcal{D}_{20}$	**.78**	134
		$\mathcal{D}_{21}$	**.83**	241
